# Neurons sensitive to non-celestial polarized light in the brain of the desert locust

**DOI:** 10.1007/s00359-023-01618-w

**Published:** 2023-02-21

**Authors:** Marius Beck, Vanessa Althaus, Uta Pegel, Uwe Homberg

**Affiliations:** 1 Department of Biology, Animal Physiology, Philipps University of Marburg, 35032 Marburg, Germany; 2 Present Address: Institute of Biology, University of Siegen, 57068 Siegen, Germany; 3 Center for Mind Brain and Behavior (CMBB), Philipps-University of Marburg and Justus Liebig University of Giessen, 35032 Marburg, Germany

**Keywords:** Non-celestial polarization vision, Central complex, Sky compass coding, Intracellular recordings, Desert locust

## Abstract

Owing to alignment of rhodopsin in microvillar photoreceptors, insects are sensitive to the oscillation plane of polarized light. This property is used by many species to navigate with respect to the polarization pattern of light from the blue sky. In addition, the polarization angle of light reflected from shiny surfaces such as bodies of water, animal skin, leaves, or other objects can enhance contrast and visibility. Whereas photoreceptors and central mechanisms involved in celestial polarization vision have been investigated in great detail, little is known about peripheral and central mechanisms of sensing the polarization angle of light reflected from objects and surfaces. Desert locusts, like other insects, use a polarization-dependent sky compass for navigation but are also sensitive to polarization angles from horizontal directions. In order to further analyze the processing of polarized light reflected from objects or water surfaces, we tested the sensitivity of brain interneurons to the angle of polarized blue light presented from ventral direction in locusts that had their dorsal eye regions painted black. Neurons encountered interconnect the optic lobes, invade the central body, or send descending axons to the ventral nerve cord but are not part of the polarization vision pathway involved in sky-compass coding.

## Introduction

Based on the incorporation and alignment of rhodopsin in microvillar membranes, the rhabdomeric photoreceptors of insects are inherently sensitive to the angle of polarization of light. As shown in several species, including flies, locust, crickets, beetles, bees, and butterflies, a specialized dorsal rim area (DRA) of the compound eye mediates the detection of polarization angle of light originating from the blue sky (Labhart and Meyer 1999; Mathejczyk and Wernet 2017). Polarization vision pathways, providing information on the sky polarization pattern include dorsal rim areas of the lamina and medulla, parts of the anterior optic tubercle, and the lateral complex in the brain. Pathways, finally, converge from both hemispheres in the central complex (CX) establishing a sky polarization compass used for spatial navigation (Homberg et al. 2011; Homberg and el Jundi 2013; Heinze 2014; Zittrell et al. 2020; Hardcastle et al. 2021).

In addition to detecting the sky polarization pattern, many insects are also sensitive to polarization angles of light reflected from shiny surfaces like bodies of water (Wildermuth 1998; Farkas et al. 2016; Obayashi et al. 2021), plant leaves (Kelber et al. 2001; Kinoshita et al. 2011), or the skin of host animals (Horváth et al. 2017; Heinloth et al. 2018). In some of these insect species, notably backswimmers, a specialized ventral eye region is particularly sensitive to horizontally polarized light reflected from the surface of ponds and lakes (Schwind 1983a, b; Heinloth et al. 2018). In horseflies, stochastically distributed ommatidial subtypes mediate the sensitivity to polarized light reflected from objects (Meglič et al. 2019). Central neural processing in all of these cases has not been explored (Heinloth et al. 2018).

Polarization vision pathways in the brain have been well studied in the desert locust *Schistocerca gregaria* (Homberg et al. 2011; el Jundi et al. 2014; Zittrell et al. 2020). Sky polarization is detected by blue-sensitive photoreceptors in a prominent DRA of the compound eye (Homberg and Paech 2002; Schmeling et al. 2014, 2015). Polarization signals are processed in the optic lobe and combined with celestial chromatic and brightness cues in neurons of the anterior optic tubercle (Pfeiffer et al. 2005; Kinoshita et al. 2007; Pfeiffer and Homberg 2007; el Jundi et al. 2011, 2014). A second pathway, possibly involved in time compensation, involves the accessory medulla and posterior optic tubercle (el Jundi and Homberg 2010). Polarization inputs from both hemispheres, finally, converge in the CX and give rise to a head-direction compass based on the direction of unpolarized sunlight and matched filter coding of sky polarization (Heinze and Homberg 2007; Pegel et al. 2018, 2019; Zittrell et al. 2020). Painting over the DRA abolishes polarization sensitivity in the sky compass network of the CX illustrating that polarization sensitivity of these neurons to zenithal stimulation originates exclusively from DRA photoreceptors of the eye (Hensgen et al. 2022).

Behavioral and electrophysiological evidence, in addition, suggests that locusts are also sensitive to polarization angles in the main parts of their eyes. Behavioral experiments showed that locusts avoid flying over polarized surfaces which might allow them to avoid large bodies of water reflecting polarized light (Shashar et al. 2005). While all photoreceptors in the DRA express blue light absorbing opsin, two types of ommatidia are distributed stochastically in the main eye (Schmeling et al. 2014). All ommatidia of the main eye contain 5 photoreceptors (R2, 3, 5, 6, 8) that coexpress blue and green opsin as well as two proximal photoreceptors (R1, 4) expressing green opsin. R7 photoreceptors express a UV opsin in type I ommatidia (65% of all ommatidia) and blue opsin in type II ommatidia (35% of ommatidia). While polarization sensitivity in the DRA is high (PS 2.4–22.4), photoreceptors in the main eye are not completely insensitive to polarization angle but, as determined for blue and green peaking receptors, have low PS values ranging from 1.9 to 2.3 (Schmeling et al. 2014). Visual interneurons in the locust brain that are distinct from the sky polarization encoding network are sensitive to polarized light at low stimulus elevations (Beetz et al. 2016). Some of these neurons showed increased polarization sensitivity when moving the stimulus from the zenith to the horizon. Painting the DRA black did not affect their polarization sensitivity indicating that photoreceptors of the main eye mediate these responses. In order to further analyze the processing of polarized light reflected from objects or water surfaces, we tested polarization sensitivity to light stimuli presented from ventral direction in locusts that had their DRAs painted black.

## Materials and methods

### Animals and preparation

Desert locusts (*Schistocerca gregaria*) were reared under crowded conditions with a 12:12 h light–dark cycle at 28 °C and 50% humidity. Experiments were performed on adult animals of both sexes at least one week after their last molt. Legs and wings were removed, leg stumps and mouthparts were fixed with dental wax and the animals were mounted vertically onto a metal holder strictly avoiding restriction of the ventral field of view. The DRA of both eyes was covered with opaque black paint (Abaddon Black, Citadel Base). The head capsule was opened anteriorly, fat tissue, tracheal air sacs and the gut were removed. Hemolymph leakage was prevented by using a thread to tie off the abdomen. To reduce body movement, the rest of the abdomen was covered with wax and, whenever necessary, contracting muscles in the head capsule were cut. A twisted and waxed metal wire was used to support the brain from the posterior side. The neural sheath was removed in the area of the CX to ease electrode penetration into neurons within or near the CX. During dissection and intracellular recording, the brain was permanently covered with locust saline (Clements and May 1974) containing 0.09 mol l^−1^ saccharose. All animal procedures were in compliance with the guidelines of the European Union (Directive 2010/63/EU) and the German Animal Welfare Act.

### Intracellular recordings and visual stimulation

**Fig. 1:**
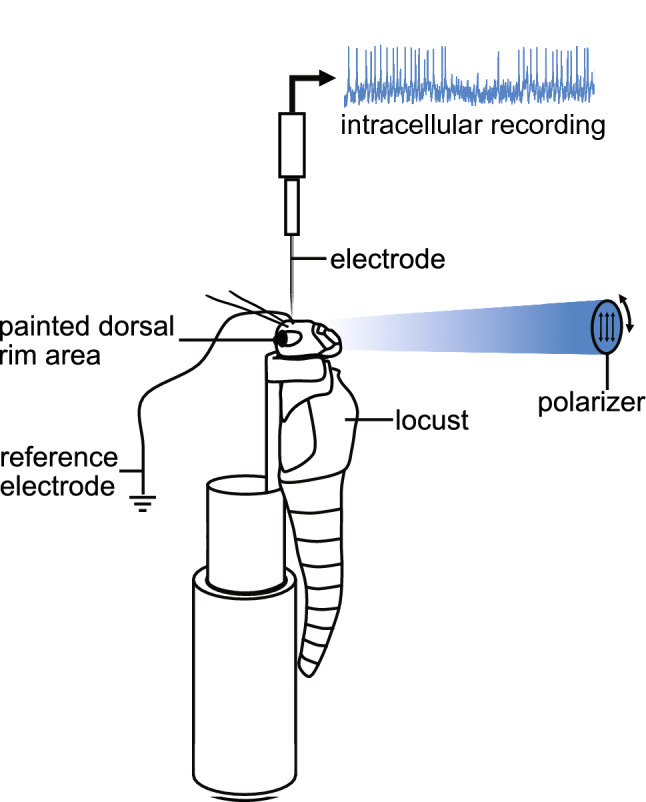
Experimental setup. The locust was mounted to a metal holder after removing legs and wings. The dorsal rim areas of both eyes were occluded with black paint. Visual stimulation was provided by a blue light-emitting diode (ELJ-465-617, 465 nm; EPIGAP Optronic, Berlin, Germany) positioned ventrally from the locust head. The stimulus directed toward the locust head was polarized by a rotatable linear polarizer (HN38S, Polaroid, Cambridge, MA, USA)

Electrodes were pulled from borosilicate glass capillaries (diameter 1.5 mm, wall thickness 0.375 mm, Hilgenberg, Malsfeld, Germany) with a Flaming/Brown horizontal puller (P97, Sutter Instrument, Novato, CA, USA). The electrode tips were filled with 4% Neurobiotin solution (Vector Laboratories, Burlingame, CA, USA), dissolved in 1 mol l^−1^ KCl and their shafts with 1 mol l^−1^ KCl. Electrode tip resistances ranged from 80 to 300 MOhm. A micromanipulator (Display SM5, Luigs & Neumann, Ratingen, Germany) was used for 3D control of the electrode. Neuronal signals were amplified 10 × via an amplifier (Sec-05x, npi electronic, Tamm, Germany) and visualized on an oscilloscope (HM507, HAMEG Instruments). The signal was digitized using an analog-to-digital converter (sampling rate: 5 kHz; Micro1401mk II, Cambridge Electronic Design, Cambridge, UK), and data were stored on a PC using Spike2 software (version 7.06; Cambridge Electronic Design). Polarized light was generated using a blue LED (ELJ-465–617, 465 nm; EPIGAP Optronic, Berlin, Germany) with an intensity of 1 × 10^14^ photons cm^−2^ s^−1^ and a motorized rotating linear polarizing filter (HN38S, Polaroid, Cambridge, MA, USA). The stimulus covered a visual angle of 3.4° and was presented to the locust’s head from the ventral direction (Fig. 1). All experiments were performed in the dark. During the recording, the polarizer was rotated 360° clockwise and counterclockwise several times at a rotational speed of 30° s^−1^. An angle of polarization parallel to the longitudinal axis of the animal was defined as 0°. Following visual stimulation, Neurobiotin was injected into the neuron by applying a constant positive current of ~ 0.5 nA for 1–5 min. After each experiment, we took a photograph of the painted eye regions, enlarged these and thereby ensured that coverage of the DRAs of both eyes had been complete. The paint could be peeled off as described by Hensgen et al. (2022). Illumination of the peeled off piece of paint placed on a digital spectrometer (USB2000, Ocean Optics, Dunedin, FL) resulted in 0 photon counts indicating that the paint was light tight.

### Histology and image processing

Brains were dissected in locust saline, immersed overnight at 4 °C in Neurobiotin fixative (4% PFA, 0.25% glutaraldehyde, and 0.2% saturated picric acid dissolved in 0.1 mol l^−1^ phosphate buffered saline (PBS)). They were stored in sodium phosphate buffer at 4 °C for up to 2 weeks. Brains were rinsed 4 × 15 min in 0.1 mol l^−1^ PBS and incubated for three days in Cy3-conjugated streptavidin (Dianova, Hamburg, Germany) diluted 1:1000 in 0.1 mol l^−1^ PBS containing 0.3% Triton X-100 (PBT) at 4 °C in the dark. Following incubation, they were rinsed 2 × in 0.1 mol l^−1^ PBT and 3 × in 0.1 mol l^−1^ PBS for 30 min each, dehydrated in an ascending alcohol series (30, 50, 70, 90, 95%, 2 × 100%) for 15 min each, and subsequently cleared in a 1:1 mixture of 100% ethanol and methyl salicylate (Merck, Darmstadt, Germany) for 20 min, followed by 100% methyl salicylate for one hour. Brains were embedded as whole mounts in Permount (Fisher Scientific, Waltham, MA, USA) between two coverslips. Preparations were scanned using a confocal laser scanning microscope (TCS SP5, Leica Microsystems, Wetzlar, Germany) with a DPSS laser (561 nm), and neurons were visualized using Amira 5.6 (ThermoFisher Scientific, Waltham, MA) or FIJI. Images were further processed, and figures were created using Adobe Photoshop and Adobe Illustrator 2021 (version 25.2.1, Adobe Systems, San José, CA, USA) and Affinity Photo (Serif Inc., Nottingham, UK). The nomenclature for brain areas follows von Hadeln et al. (2018) and for neuronal cell types of the CX, Heinze and Homberg (2008) and von Hadeln et al. (2020).

### Physiological data analysis

The physiological data were visualized using Spike2 and exported as a mat-file. Mean spiking frequencies were calculated and displayed using the Spike2 implementation of a moving average (window size 0.5 s). All further analyses were performed with custom functions written in MATLAB (version 2021a, The MathWorks, Natick, MA, USA). Action potentials were detected using a threshold. Discrete mean spike activity was calculated for all stimulus presentations (i.e., 360° rotation of the polarizer) in 10° bins to generate a stimulus–response curve. Mean responses for the two stimulus regimes (i.e., clockwise and counterclockwise rotation of the polarizer) were calculated by averaging all respective single trials and doubling the angular variable. A modulation of spiking activity dependent on angle of polarization was determined by linear-circular correlation analysis for both, single trials and calculated mean responses (Zar 2010). The angles of bin centers were used as the angular variable and the mean spiking activity in each bin as the linear variable. Responses to the rotating polarizer were tested for bidirectionality; thus, the angular variable was doubled (Batschelet 1981). The responsiveness of a neuron to the plane of polarized light was indicated by the significance (*α* ≤ 0.05) of the correlation coefficient (*r*). Its square, the coefficient of determination (*r*^2^), indicates the strength of the correlation (CS) between spike rate and angle of polarization. The length of the mean vector *|r|* quantifies vector strength (VS). It was used as a measure of response amplitude as introduced for central-complex neurons by Bockhorst and Homberg (2015) and ranges from 0 to unity. In neurons responsive to the angle of polarized light the preferred angle of polarization (*Φ*_max_) was calculated. Spike times were transformed into angles by multiplying them with the rotation velocity of the stimulus. Angles were doubled and the mean angle calculated (Batschelet 1981), indicating the angle of polarization the neuron is tuned to (*Φ*_max_). The angle orthogonal to *Φ*_max_ was regarded as the anti-preferred angle *Φ*_min_. Differences in tuning to clockwise vs. counterclockwise rotation of the polarizer are indicated as Δ*Φ*_max_ = *Φ*_max_ clockwise–*Φ*_max_ counterclockwise. The background activity of neurons was determined using the areas of the spike train in which no rotational stimulus was presented and lights turned off. Action potentials in these areas were counted, and the mean spike rate per second was calculated.

## Results

We recorded from 43 neurons in the *S. gregaria* brain, including 6 neurons with arborizations in the optic lobe, 31 CX neurons, and 5 descending neurons. We examined the responses for sensitivity to the angle of polarization of blue light presented from ventral direction. Eleven neurons showed polarization sensitivity during both directions of rotation of the polarizer and 9 additional neurons during one rotation direction.

### Neurons of the optic lobe

**Fig. 2:**
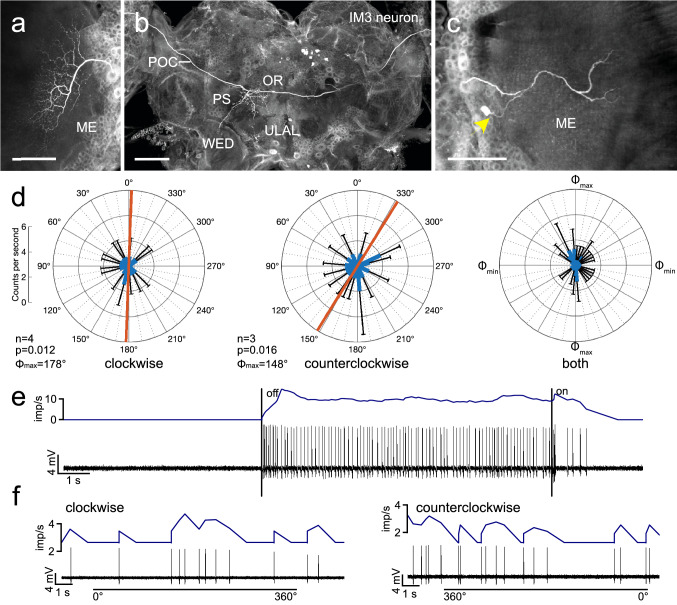
Morphology and physiology of intermedulla neuron IM3. **a–c** Projection views of the Neurobiotin-labeled IM3 neuron. The neuron has ipsilateral ramifications in a small equatorial sector of an outer medulla (ME) layer. An axonal fiber crosses the brain midline in the posterior optic commissure (POC) and gives rise to beaded side branches in the posterior slope (PS), which partly extend into the wedge (WED), ocellar root (OR), and upper lateral accessory lobe (ULAL). The axon continues toward the contralateral optic lobe and gives rise to wide-field ramifications in an outer layer of the ME. Yellow arrowhead in **c** points at the soma near the accessory medulla. **d** Circular diagrams showing mean spiking activities (+ SD, black bars) plotted against polarization angle during 4 rotations in clockwise (left) and 3 rotations in counterclockwise (middle) direction. Red bars indicate preferred polarization angle (*Φ*_max_). Right diagram shows mean activities (+ SD) from all rotations with *Φ*_max_ set at 0°. **e** Spike train (bottom trace) and mean spiking activity illustrating changes in activity when turning the light source (blue LED, polarizer at 0°) off and on. **f** Spike trains (bottom traces) and mean spike frequency (top, moving average with bin size of 0.5 s) during clockwise (0°–360°) and counterclockwise (360°–0°) rotation of the polarizer. Scale bars = 150 µm

**Fig. 3:**
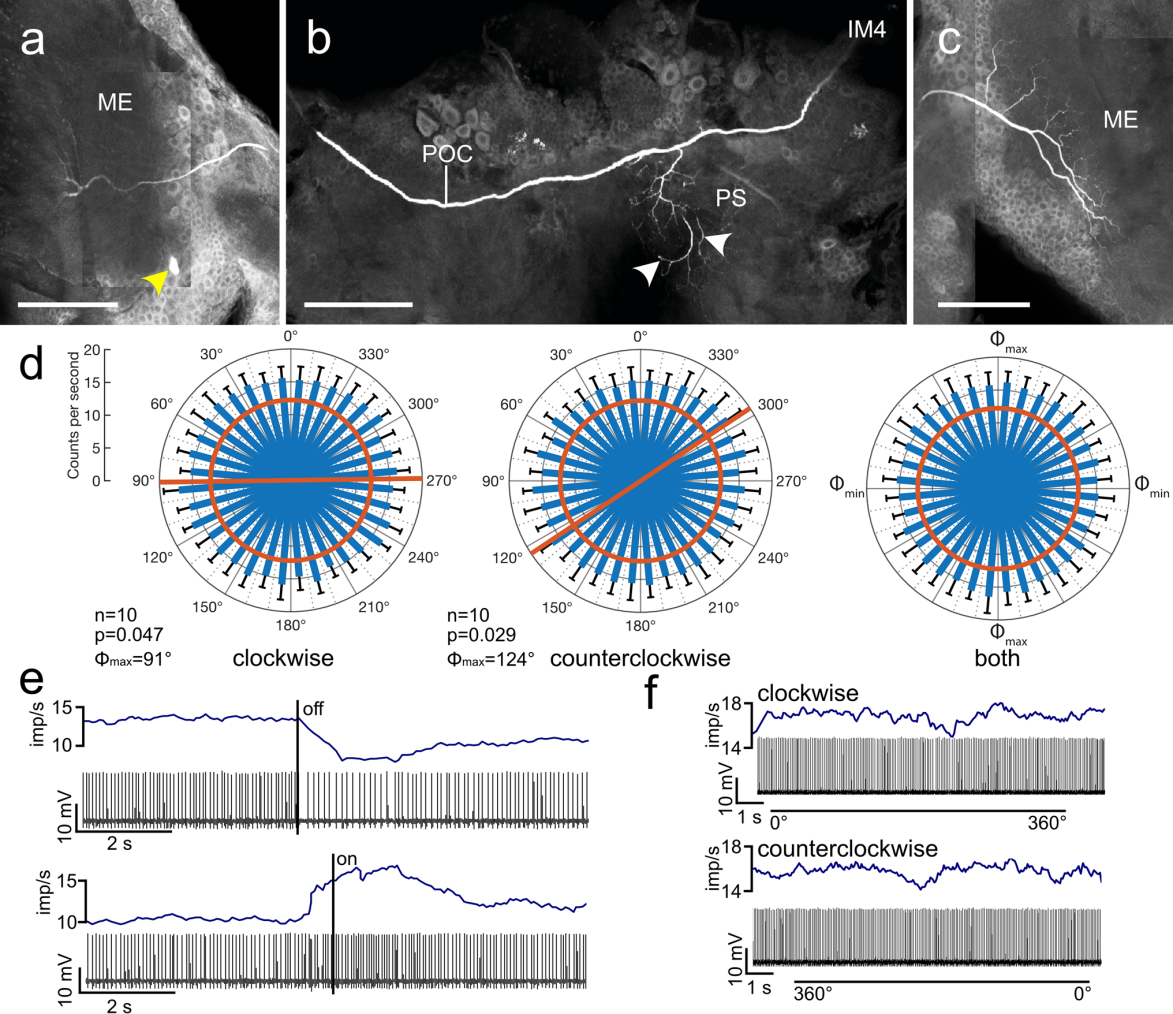
Morphology and physiology of intermedulla neuron IM4. **a-c** Stacks of confocal images of the Neurobiotin-labeled IM4 neuron. **a** The neuron has ipsilateral ramifications in a small equatorial sector of an outer medulla (ME) layer. Yellow arrowhead points at the soma near the accessory medulla. **b** An axonal fiber crosses the brain midline in the posterior optic commissure (POC) and gives rise to beaded side branches in the posterior slope (PS, arrowheads). **c** The axon continues toward the contralateral optic lobe and gives rise to wide-field ramifications in an outer layer of the ME. **d** Circular diagrams showing mean spiking activities (+ SD, black bars) plotted against polarizer orientation during 10 rotations in clockwise (left) and counterclockwise (middle) direction. Red bars indicate preferred polarization angle (*Φ*_max_), red circles indicate background activity in the dark. Right diagram shows mean activities (+ SD) from all rotations with *Φ*_max_ set at 0°. **e** Spike train (bottom trace) and mean spiking activity (top trace, moving average with bin size of 0.5 s) illustrating changes in activity when turning the light source (blue LED, polarizer set at 0°) off and on. **f** Spike trains (bottom traces) and mean spike frequency (top, moving average with bin size of 0.5 s) during clockwise (0°–360°) and counterclockwise (360°–0°) rotation of the polarizer. Scale bars = 200 µm

Recordings were obtained from three neurons connecting the medulla of both hemispheres, termed intermedulla neurons (IM) and three neurons with more complex ramifications ipsilaterally in the lobula complex and contralaterally in the lobula complex and medulla. Those neurons were termed interlobular-medulla neurons (ILM). The IM neurons showed morphological similarities with two previously characterized IM neurons (Beetz et al. 2016), but because of distinct differences from the earlier characterized cells were termed IM3 and IM4. IM3- and IM4 neurons connected the right and left medulla and had additional beaded ramifications in the contralateral posterior slope of the central brain (Figs. 2a–c, 3a–c). The soma of IM3 was located near the accessory medulla anterior-medial to the medulla (Fig. 2c). Fine processes innervated a small sector near the equator in an outer layer of the ipsilateral medulla (Fig. 2c). The main neurite passed through the posterior optic commissure into the contralateral brain hemisphere and into the contralateral optic lobe (Fig. 2a, b). In the contralateral optic lobe, the neuron ramified widely in an outer layer of the medulla (Fig. 2b), possibly layer 4 as defined by Rosner et al. (2017). In the contralateral hemisphere of the central brain beaded side branches were given off into the posterior slope. Some of these extended into the ocellar root, the lateral accessory lobe, and the wedge (Fig. 2b). The neuron had a background activity of 11.7 ± 3.6 (SD) imp s^−1^ and was strongly inhibited by the blue light with a stationary angle of polarization of 0° (Fig. 2e), as well as during all other polarizer orientations. Correlation analysis revealed a significant dependence of the response to the angle of polarization, i.e., reduced inhibition at *Φ*_max_ in both directions of polarizer rotation (Fig. 2d, f, Table 1). The preferred angle of polarization was at *Φ*_max_ = 178° for clockwise and at *Φ*_max_ = 148° for counterclockwise polarizer rotation, respectively (Fig. 2d). The rotation-direction dependent shift in tuning of the neuron was Δ*Φ*_max_ = 30°.

**Table 1:** Sensitivity to polarization angle of light from ventral direction in the desert locust

Cell type	Clockwise rotation					Counterclockwise rotation					Figures
	*n*^a^	*Φ*_max_^b^	*p*^c^	VS/*|r|*^d^	CS/*r*^*2*e^	*n*^a^	*Φ*_max_^b^	*p*^c^	VS/*|r|*^d^	CS/*r*^*2*e^	
IM3	4	178°	**0.012**	0.102	0.246	3	148°	**0.016**	0.160	0.231	Figure 2
IM4	10	91°	**0.047**	0.010	0.17	10	124°	**0.029**	0.012	0.196	Figure 3
IM4	7	129°	**0.012**	0.020	0.246	7	n.s.	0.08	0.012	0.139	
ILM	10	69°	**0.001**	0.071	0.392	11	102°	**8 × 10**^**–5**^	0.095	0.527	Figure 4
ILM	7	122°	**0.01**	0.020	0.224	7	148°	**0.013**	0.019	0.241	
ILM	2	50°	**0.018**	0.141	0.224	2	n.s.	0.219	0.071	0.084	
CL1	2	n.s.^f^	0.133	0.064	0.112	1	n.s.	0.083	0.096	0.138	
CL1	3	n.s.	0.132	0.031	0.113	3	n.s.	0.271	0.025	0.073	
CL1	2	n.s.	0.322	0.029	0.063	2	114°	**0.031**	0.050	0.193	
CL1	4	124°	**0.002**	0.114	0.356	4	n.s.	0.268	0.036	0.073	Figure 5
CL1	1	n.s.	0.69	0.038	0.021	1	n.s.	0.09	0.097	0.133	
CPU1a	2	161°	**0.005**	0.104	0.295	2	n.s.	0.366	0.017	0.056	Figure 6a, b
CPU1a	4	n.s.	0.627	0.003	0.026	4	n.s.	0.136	0.009	0.111	
CPU1a	7	116°	**0.007**	0.022	0.275	7	n.s.	0.921	0.001	0.005	
CPU1a	10	n.s.	0.156	0.042	0.103	8	n.s.	0.843	0.003	0.009	
CPU1a	2	n.s.	0.473	0.011	0.042	2	n.s.	0.329	0.024	0.062	
CPU2	4	n.s.	0.836	0.006	0.01	4	n.s.	0.614	0.014	0.027	
CPU3	4	n.s.	0.205	0.044	0.088	4	n.s.	0.687	0.017	0.021	
CPU5	3	n.s.	0.633	0.015	0.025	3	n.s.	0.422	0.020	0.048	
CPU5	3	59°	**0.002**	0.176	0.352	3	33°	**0.049**	0.109	0.168	Figure 6c–e
CU	3	n.s.	0.493	0.029	0.039	3	n.s.	0.369	0.034	0.055	
TU_SLP_4	2	18°	**0.034**	0.231	0.188	3	18°	**0.049**	0.130	0.168	Figure 7a–d
TL2	9	87°	**0.045**	0.167	0.173	9	n.s.	0.428	0.040	0.047	Figure 7e, f
TB1b	3	n.s.	0.27	0.024	0.072	3	n.s.	0.192	0.053	0.092	
TB1b	3	n.s.	0.352	0.012	0.058	3	n.s.	0.24	0.018	0.079	
TB1c	3	n.s.	0.667	0.011	0.025	3	n.s.	0.692	0.009	0.204	
TB1d	5	n.s.	0.653	0.010	0.024	5	n.s.	0.457	0.015	0.044	
TB1d	2	n.s.	0.957	0.010	0.002	2	n.s.	0.508	0.026	0.038	Figure 8a, b
TB2	5	n.s.	0.552	0.007	0.033	5	n.s.	0.758	0.009	0.015	Figure 8c, d
TB3	4	n.s.	0.387	0.022	0.052	3	n.s.	0.208	0.029	0.087	
POU1	3	49°	**0.021**	0.358	0.215	9	43°	**7 × 10**^**–4**^	0.383	0.633	Figure 9
POU1	3	n.s.	0.386	0.093	0.052	2	84°	**0.011**	0.231	0.251	
POU2	3	n.s.	0.935	0.035	0.004	3	n.s.	0.981	0.015	0.001	
POU3	9	n.s.	0.446	0.014	0.045	6	n.s.	0.597	0.016	0.029	
POU3	2	n.s.	0.150	0.034	0.105	1	n.s.	0.939	0.017	0.003	
POU3	3	n.s.	0.276	0.051	0.071	3	n.s.	0.17	0.065	0.099	
POU3	7	n.s.	0,834	0.005	0.010	6	n.s.	0.499	0.009	0.039	
ILP	6	71°	**0.007**	0.048	0.274	6	98°	**0.016**	0.062	0.23	Figure 10
PI(2):5	3	n.s.	0.131	0.143	0.113	2	n.s.	0.053	0.307	0.163	
PI(2):6	6	96°	**9.9 × 10**^**–7**^	0.065	0.768	6	119°	**1 × 10**^**–4**^	0.036	0.499	Figure 11
PI(2):6	4	56°	**0.005**	0.062	0.292	3	80°	**2 × 10**^**–4**^	0.112	0.469	
IDN1	6	158°	**0.039**	0.025	0.181	7	151°	**0.01**	0.024	0.257	Figure 12
IDN2	16	n.s.	0.201	0.009	0.089	16	n.s.	0.166	0.007	0.1	Figure 13

^a^*n* = number of rotations

^b^*Φ*_max_ = preferred angle of polarization

^c^*p* = significance of correlation coefficient (*p* values < 0.05 are shown in bold)

^d^VS/*|r|*= vector strength (VS) defined by the length of the mean vector *|r|*

^e^CS/*r*^*2*^ = correlation strength (CS) defined by the coefficient of determination *r*^*2*^

^f^n.s. = not significant

Two recordings were from IM4 neurons (Fig. 3). The IM4 neurons were morphologically highly similar to the IM3 neuron, but had less extensive ramifications in the central brain confined to the posterior slope (Fig. 3b) and showed a slight response distinct from that of IM3. Background activity was 12.4 ± 1.0 (SD) imp s^−1^ (Fig. 3) and 6.6 ± 0.0 (SD) imp s^−1^. In contrast to IM3, both IM4 neurons were excited by ventral blue light irrespective of polarizer orientation (Fig. 3d, e). One of the two neurons showed a weak but significant maximum excitation at particular angles of polarization in both directions of rotation with Δ*Φ*_max_ = –33° (clockwise: *Φ*_max_ = 91°, counterclockwise: *Φ*_max_ = 124°; Fig. 3d, f). The second neuron showed a significant tuning to polarization angle only during clockwise rotation of the polarizer (*Φ*_max_ = 129°; Table 1).

**Fig. 4:**
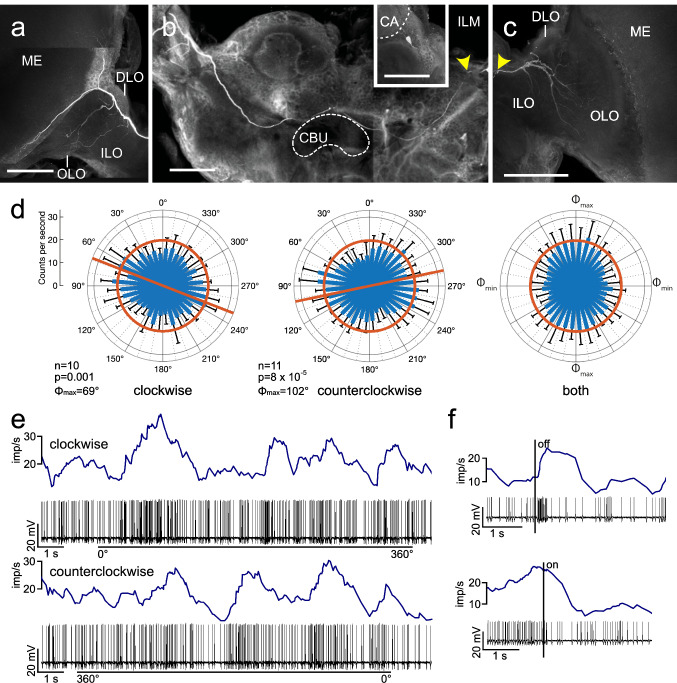
Morphology and physiology of the interlobula-medulla neuron (ILM). **a**–**c** Stacks of confocal images of the neuron. **c** In the ipsilateral hemisphere, the neuron innervates the inner (ILO) and outer lobe (OLO) of the lobula complex. **b** Its axon crosses the brain midline in the superior arch commissure dorsally from the upper division of the central body (CBU). Inset shows cell body of the neuron near the calyx (CA) of the mushroom body. Yellow arrowheads in **b** and **c** point at the site where the cell body fiber joins the main neurite of the neuron. **a** In the contralateral optic lobe, the neuron targets the ILO, OLO, and dorsal lobe (DLO) of the lobula complex and has continuing fibers into the medulla (ME). **d** Circular plots showing mean spiking activity (+ SD, black bars) plotted in 10° bins during clockwise and counterclockwise rotation of the polarizer. Red bars indicate preferred polarization angles, red circles indicate background activity in the dark. Right diagram shows mean activities (+ SD) from all rotations with *Φ*_max_ set at 0°. **e** Spike trains (bottom traces) and mean spike frequency (top, moving average with bin size of 0.5 s) during clockwise (0°–360°) and counterclockwise (360°–0°) rotation of the polarizer. **f** Change in spike rate when switching the light source (blue LED, polarizer at 0°) off and on. Scale bars = 200 µm

Three recordings were made from ILM neurons. The neurons were morphologically indistinguishable. Their somata were located in the cell body rind above the superior lateral protocerebrum, near the mushroom-body calyx (Fig. 4b inset). Fine branches innervated the outer and inner lobe of the ipsilateral lobula complex (Fig. 4c). Axonal fibers crossed the brain midline in the superior arch commissure along the dorsal face of the upper division of the central body (CBU, Fig. 4b). In the contralateral optic lobe, the neurons innervated the dorsal, outer and inner lobe of the lobula complex. A fiber bypassing the lobula complex bifurcated at the anterior inner margin, one collateral passing dorsally and the other one ventrally along the inner face of the medulla (Fig. 4a). Both collaterals gave rise to ramifications in an inner layer of the medulla, likely layer 9 and/or 10. No side branches were observed in the central brain (Fig. 4b). Background activities of the ILM neurons were 9.9 ± 2.0 (SD) imp s^−1^, 24.4 ± 6.6 (SD) imp s^−1^ and 18.2 ± 3.1 (SD) imp s^−1^. The neurons were transiently inhibited by switching on the blue light with stationary polarizer at 0° and showed a short phasic excitation following lights off (Fig. 4f). Two of the three ILM neurons showed preferred angles of polarization, i.e. minimum inhibition but no net excitation, during both directions of rotation of the polarizing filter (Fig. 4d, e; Table 1), whereas the third cell showed significant angle of polarization tuning only during clockwise rotation of the polarizer rotation (Table 1). The preferred polarizer angles differed in the three neurons suggesting that recordings originated from different individual cells of the same morphological type.

### Central-complex neurons

The central complex (CX) is specifically targeted by polarization vision pathways from the DRAs of both eyes transmitting celestial polarization information (Homberg et al. 2003, 2011). Computations within the CX result in a compass-like representation of spatial directions in the protocerebral bridge (PB) of the CX (Heinze and Homberg 2007; Pegel et al. 2019; Zittrell et al. 2020). We were, therefore, interested in exploring the possibility that the CX might also serve as a site for evaluating non-celestial polarization information. In total, recordings were obtained from 31 neurons with arborizations in the CX, including 15 columnar neurons, 9 tangential neurons, and 7 pontine neurons.

#### Columnar neurons

**Fig. 5:**
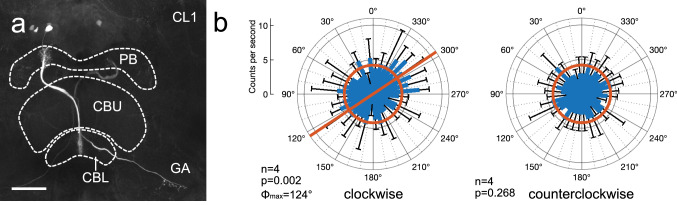
Tuning to polarization angle of light from ventral direction in a CL1 neuron of the central complex. **a** The neuron innervates column 4 in the right hemisphere of the protocerebral bridge (PB), the innermost column 1 in the right hemisphere of the lower division of the central body (CBL), and sends an axon to the gall (GA). **b** Circular plots showing mean spiking activity (+ SD, black bars) plotted in 10° bins during clockwise and counterclockwise rotation of the polarizer. Red bar in the left diagram indicates preferred polarization angle, red circles indicate background activity in the dark. No significant tuning to polarization angle was observed during counterclockwise rotation of the polarizer. Scale bar = 100 µm

Five CL1-type columnar neurons with arborizations in the protocerebral bridge (PB) and lower division of the central body (CBL) could be examined for responsiveness to polarized light from ventral direction (Table 1). Their somata were located in the pars intercerebralis. The neurons connected ipsilateral columns of the PB to specific columns of the CBL. Small axonal fibers extended to the lateral accessory lobe and innervated the gall with beaded ramifications (Fig. 5a). Background activities of the neurons ranged from 4.2 ± 2.5 (SD) imp s^−1^ to 12.3 ± 0.3 (SD) imp s^−1^. Two of the five CL1 neurons showed a directional response to the rotating polarizer, however only during one of the two turning directions. In one cell, *Φ*_max_ during clockwise rotation was 124° (Fig. 5b), whereas no significant tuning to polarization angle was found when the polarizing filter was rotated counterclockwise (Fig. 5b). The second neuron showed significant angular tuning during counterclockwise rotation of the polarizer with a preferred angle of polarization at 114° (Table 1), but was insensitive to the angle of polarization during clockwise rotation of the polarizer (Table 1).

**Fig. 6:**
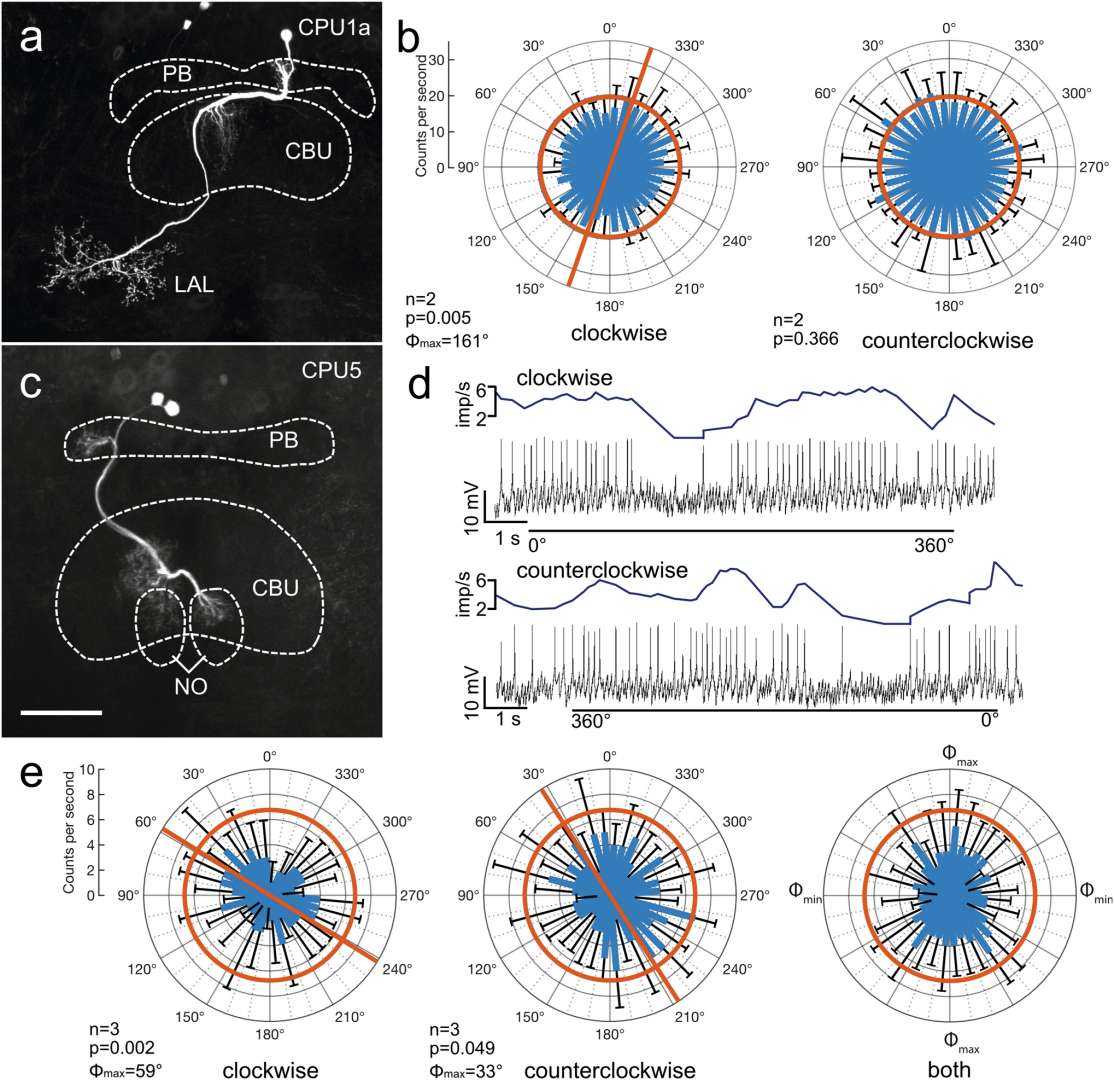
Sensitivity to polarization angle in two types of columnar neuron of the upper division of the central body (CBU). **a**, **b** Morphology and physiology of a type 1a columnar neuron of the CBU (CPU1a). The neuron has columnar ramifications in the protocerebral bridge (PB) and the CBU and sends an axon with beaded terminals into the lateral accessory lobe (LAL). **b** Circular plots showing mean spiking activity (+ SD, black bars) plotted in 10° bins during clockwise and counterclockwise rotation of the polarizer. Red bar in **b** indicates preferred polarization angle. No tuning to polarization angle was found during counterclockwise polarizer rotation. Red circles indicate background activity in the dark. **c–e** Morphology and physiology of a type 5 columnar neuron of the CBU (CPU5). **c** The neuron has columnar ramifications in the PB and the CBU and in the upper unit of the contralateral nodulus (NO). **d** Spike trains (bottom traces) and mean spike frequency (top, moving average with bin size of 0.5 s) during clockwise (0°–360°) and counterclockwise (360° − 0°) rotation of the polarizer. **e** Circular plots showing mean spiking activity (+ SD, black bars) plotted in 10° bins during clockwise and counterclockwise rotation of the polarizer. Red bars indicate preferred polarization angles. Throughout both rotation directions spiking activity of the neuron is reduced compared to background activity in the dark (red circles). Diagram on the right shows mean activities (+ SD) from all rotations with *Φ*_max_ set at 0°. Scale bars = 200 µm (**a**), 100 µm (**c**)

In five recordings CPU1a-type columnar neurons were examined for responsiveness to ventrally presented polarized light. The somata of the cells were located in the pars intercerebralis. The neurons connected an ipsilateral column of the PB to certain columns of the upper division of the central body (CBU). From there, an axon projected to wide beaded ramifications in the contralateral lateral accessory lobe (Fig. 6a). Background activities ranged from 19.4 ± 14.1 (SD) imp s^−1^ to 38.3 ± 5 (SD) imp s^−1^. The neurons were slightly excited by blue light from ventral direction with stationary angle of polarization of 0°. In two of the five CPU1 neurons, clockwise rotation of the polarizer revealed responses to the ventral polarized light stimulus. One cell had a preferred *Φ*_max_ of 161° (Fig. 6b), whereas the preferred polarization angle of the second cell was at a *Φ*_max_ of 116° (Table 1).

Two recordings were from CPU5-type columnar neurons. Their somata were located in the pars intercerebralis. The neurons connected single columns of the PB with several columns in layer III of the CBU, and invaded dorsal layers of the upper unit of the contralateral nodulus (Fig. 6c). Background activities of the neurons were 6.7 ± 0.5 imp s^−1^ (Fig. 6c) and 15.5 ± 6.9 imp s^−1^. Spiking activity in both neurons was reduced during ventral light stimulation (Fig. 6e). One neuron was sensitive to the angle of polarization during both rotation directions, with a more pronounced response during clockwise than during counterclockwise rotation (clockwise: *Φ*_max_ = 59°, counterclockwise: *Φ*_max_ = 33°; Fig. 6d, e), and an angular difference in tuning between both rotation directions of Δ*Φ*_max_ = 26°. The second neuron was insensitive to polarization angle during ventral stimulation (Table 1).

Recordings from three other types of columnar neurons showed no sensitivity to the polarization angle during stimulation from ventral direction (Table 1). These were a CPU2 neuron with axonal projections to both lateral accessory lobes, a CPU3 neuron without axonal projections to the lateral accessory lobe, and a double impalement of two CU neurons, likely CU1 or CU3. These neurons had columnar ramifications in the CBU and continuing processes innervating the anterior lip and both lateral accessory lobes.

#### Tangential neurons

**Fig. 7:**
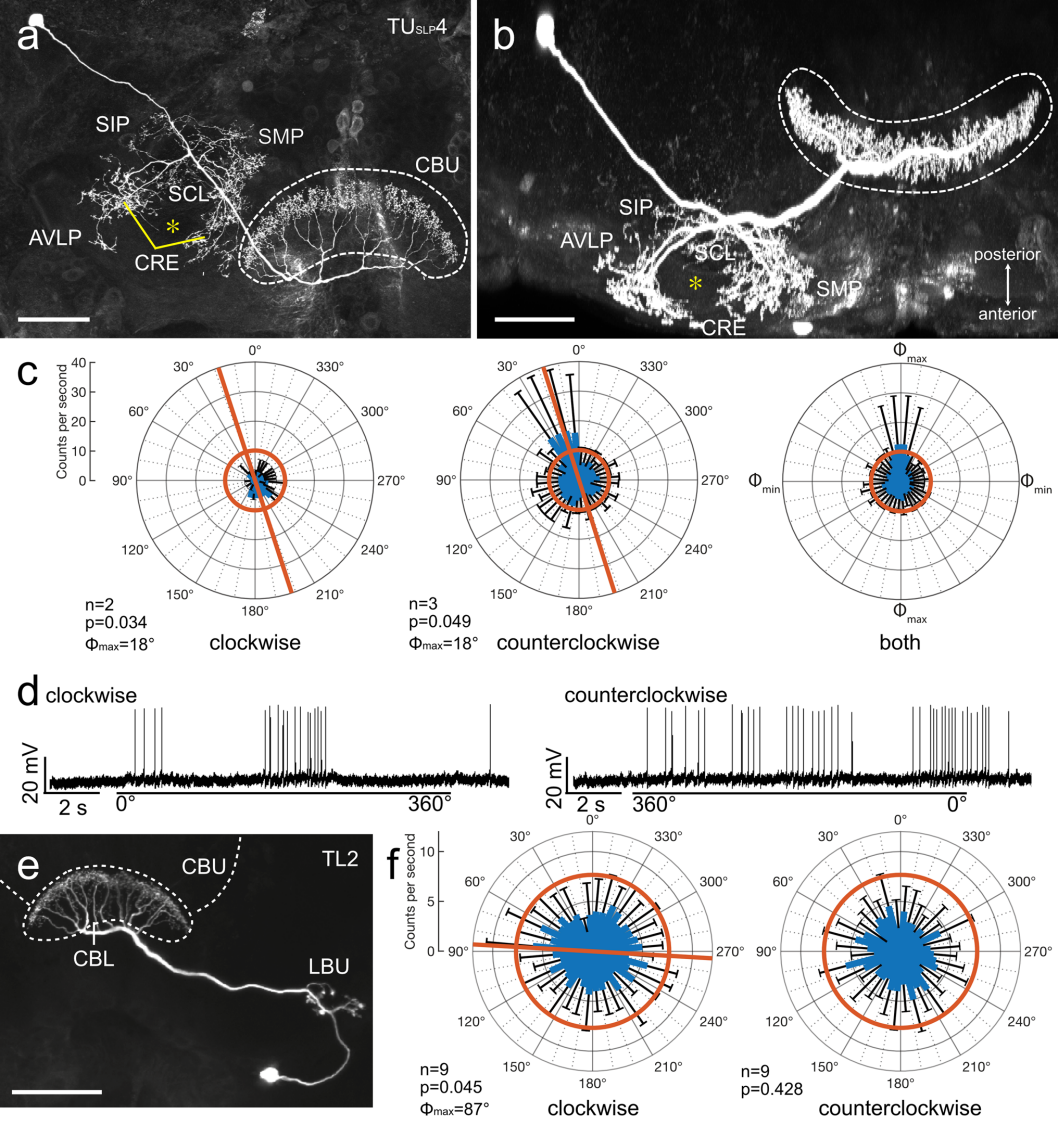
Sensitivity to polarization angle in two tangential neurons of the central body. **a-d** Morphology and physiology of a type 4 tangential neuron of the upper division of the central body (CBU) with soma near the superior lateral protocerebrum (TU_SLP_4). The neuron has wide ramifications in several brain areas, including the superior medial protocerebrum (SMP), the superior intermediate protocerebrum (SIP), the crepine (CRE), the anterior ventro-lateral protocerebrum (AVLP), and the superior clamp (SCL) encircling the vertical lobe of the mushroom body (asterisks in **a** and **b**). Its axon enters the central body through the anterior bundle and gives rise to beaded ramifications throughout layer IIa of the CBU. **c** Circular plots showing mean spiking activity (+ SD, black bars) plotted in 10° bins during clockwise (left) and counterclockwise rotation of the polarizer (middle). Red bars indicate preferred polarization angles. Red circles indicate background activity in the dark. Diagram on the right shows mean activities (+ SD) from all rotations with *Φ*_max_ set at 0°. **d** Spike trains during clockwise (0°–360°) and counterclockwise (360° − 0°) rotation of the polarizer. **e** Morphology and physiology of a TL2 neuron connecting the lateral bulb (LBU) and lower division of the central body (CBL). **f** Circular plots showing mean spiking activity (+ SD, black bars) plotted in 10° bins during clockwise and counterclockwise rotation of the polarizer. Red bar indicates preferred polarization angle. Red circles indicate background activity in the dark. Scale bars = 100 µm

Recordings were obtained from 9 tangential neurons of the CX including one neuron innervating the CBU, one neuron with ramifications in the CBL, and seven neurons innervating the PB. One neuron of the TU_SLP_4 subtype was studied (Fig. 7a–d). The soma of the cell was located ventrolateral to the calyx in the superior lateral protocerebrum. The main neurite of the cell passed through the anterior bundle toward the CBU. Fine, likely dendritic side branches were concentrated around the vertical lobe of the mushroom body in a wide mesh of various neuropils (Fig. 7a, b). The main fiber entered the central body through the second branch of the anterior bundle and gave rise to fan-like ramifications in layer IIb of the CBU (Fig. 7a, b). The background activity was 8.1 ± 5.4 (SD) imp s^−1^. The neuron was tuned to a polarization angle of *Φ*_max_ = 18° during both directions of polarizer rotation (Fig. 7c, d; Table 1).

A TL2 tangential neuron arborizing in the CBL was tested for polarization sensitivity from ventral direction. Its soma was located ventral to the lateral accessory lobe. The neuron innervated the lateral bulb. An axonal fiber passed via the isthmus tract to the CBL and gave rise to wide fan-like arborizations in layer 2 of the CBL (Fig. 7e). The neuron had a background spiking activity of 7.7 ± 2.0 (SD) imp s^−1^ and was inhibited by ventral illumination of the locust eyes. The neuron showed a weak polarization angle-dependent response when the polarizer was rotated clockwise (*Φ*_max_ = 87°; Fig. 7f), but was insensitive to polarization angle when the polarizer was rotated in counterclockwise direction (Table 1).

**Fig. 8:**
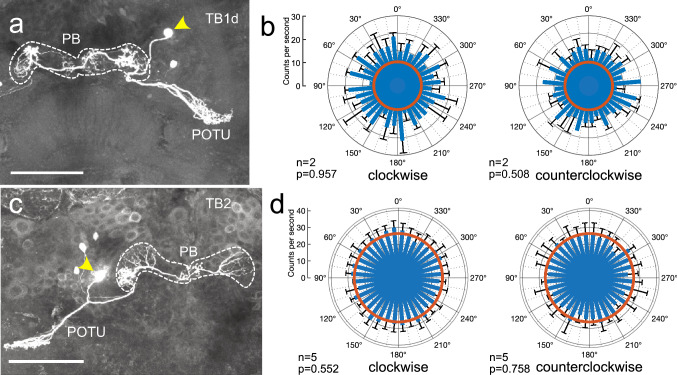
Sensitivity to polarization angle in two tangential neurons of the protocerebral bridge (PB). Both neurons connect the posterior optic tubercle (POTU) with the PB but differ in the innervated columns of the PB. Arrowheads in **a** and **c** point to soma position of the neurons. **a** Morphology of a TB1d tangential neuron with varicose ramifications in columns 5 in the ipsilateral and 4 in the contralateral hemisphere of the PB. **b** Circular plots from the neuron in **a** showing mean spiking activity (+ SD, black bars) plotted in 10° bins during clockwise rotation (left) and counterclockwise rotation of the polarizer (right). The neuron shows no angular preference to polarized light. Red circles indicate background activity in the dark. **c** Morphology of a TB2 tangential neuron with varicose ramifications in columns 1 and 8 in the ipsilateral and 1 in the contralateral hemisphere of the PB. **d** Circular plots from the neuron in **c** showing mean spiking activity (+ SD, black bars) plotted in 10° bins during clockwise rotation (left) and counterclockwise rotation of the polarizer (right). The neuron shows no angular preference. Red circles indicate background activity in the dark. Scale bars = 100 µm

Seven tangential neurons of the PB were studied, including 5 TB1-type neurons (Fig. 8a), one TB2-type neuron (Fig. 8c) and one TB3-type neuron. The TB1 neurons were of the subtype TB1b (two neurons), TB1c (one neuron; Fig. 8a) and TB1d (2 neurons) as characterized by Beetz et al. (2015). All studied TB neurons innervated the posterior optic tubercle and the PB. TB1 neurons have varicose ramifications in one column of the PB in each hemisphere, TB2 neurons, in the outermost column of one hemisphere and in the innermost columns of both hemispheres, and TB3 neurons only invade the ipsilateral hemisphere of the PB. TB1 neurons had background activities ranging from 5.3 ± 8.9 (SD) imp s^−1^ to 23.4 ± 4.5 imp s^−1^. All TB neurons were insensitive to polarization angle (Fig. 8, Table 1).

#### Pontine neurons

**Fig. 9:**
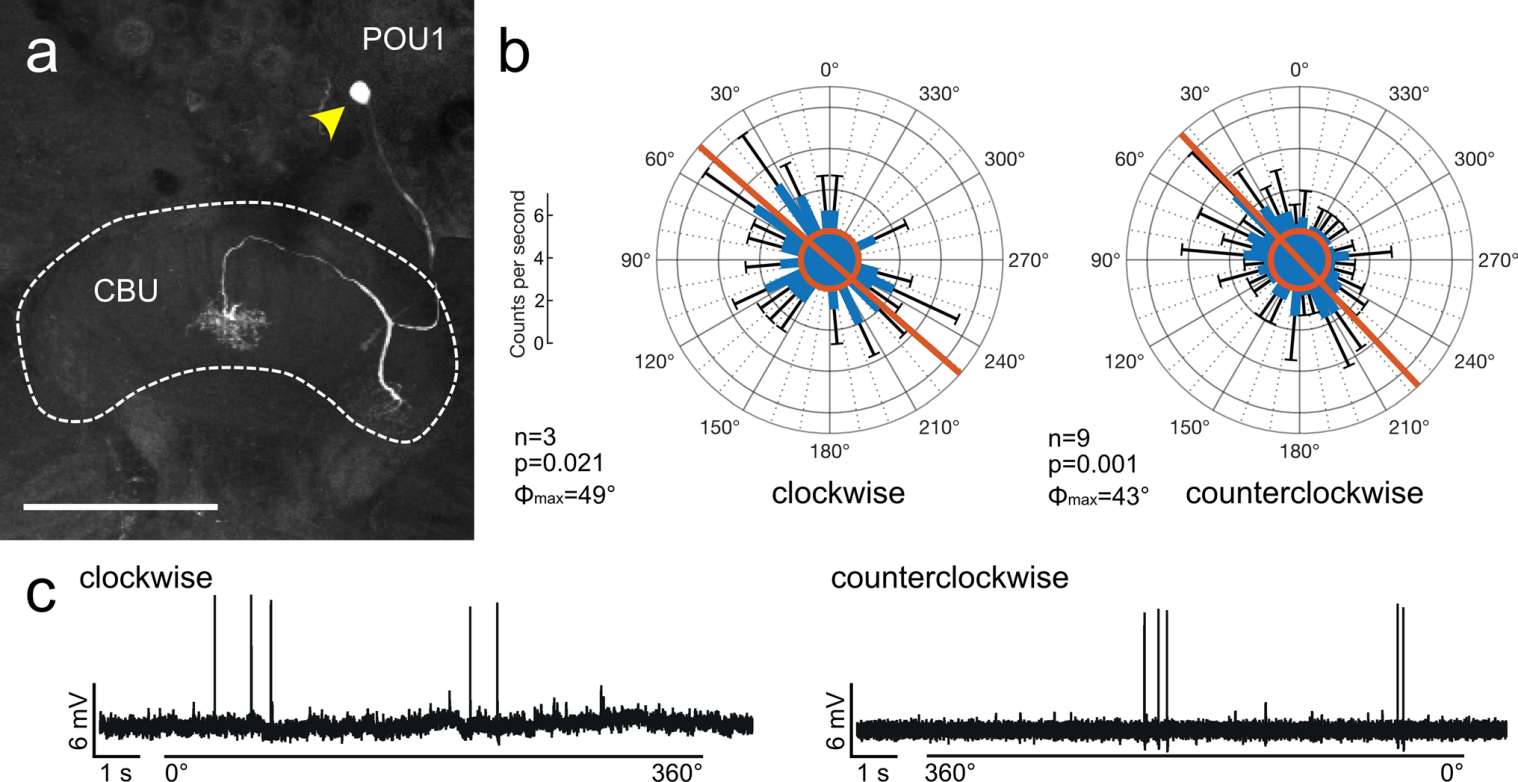
Sensitivity to polarization angle in a POU1 pontine neuron of the upper division of the central body (CBU). **a** Stack of optical sections showing the morphology of the neuron. It has fine arborizations in outermost ipsilateral columns of the CBU and a second field of finely beaded processes in innermost contralateral columns. Yellow arrowhead indicates the position of the cell body. **b** Circular plots showing mean spiking activity (+ SD, black bars) plotted in 10° bins during clockwise rotation (left) and counterclockwise rotation of the polarizer (right). Red circles indicate background activity in the dark. The neuron shows increased spiking activity during polarizer rotation in both directions. Red bars indicate preferred polarization angles. **c** Spike trains during clockwise (0°–360°) and counterclockwise (360° − 0°) rotation of the polarizer. Scale bar = 150 µm

Seven pontine neurons (2 POU1, 1 POU2, 4 POU3) were studied. Pontine neurons connect individual columns of the CBU that are 8 columns apart. Depending on the innervation layers POU1, 2 and 3 neurons have been distinguished (Heinze and Homberg 2008). The somata of the neurons were located in the pars intercerebralis. Background activity was low in the POU1 neurons (2.4 ± 1.7 and 0.0 ± 0.0 imp s^−1^), slightly higher in the POU2 neuron (8 ± 0.9 imp s^−1^), and highest in POU3 neurons (9.4 ± 4.5, 11.1 ± 4.5 and 11.9 ± 5.5 imp s^−1^). All POU neurons except for two POU3 neurons were slightly excited by the blue light irrespective of polarizer orientation (Fig. 9b). When presenting light through a stationary polarizer (angle of polarization 0°) the activity increase was phasic and decreased again over time. One POU1 neuron showed polarization angle dependent responses in both directions of polarizer rotation (clockwise: *Φ*_max_ = 49°, counterclockwise: *Φ*_max_ = 43°, Δ*Φ*_max_ = 6°; Fig. 9a, b). The second POU1 neuron was sensitive to polarization angle only during counterclockwise rotation of the polarizer (*Φ*_max_ = 84°; Table 1). The recordings from POU2 and POU3 subtypes showed no angle of polarization dependence in their responses to the rotating polarizer (Table 1).

### Interneuron of the cerebrum

**Fig. 10:**
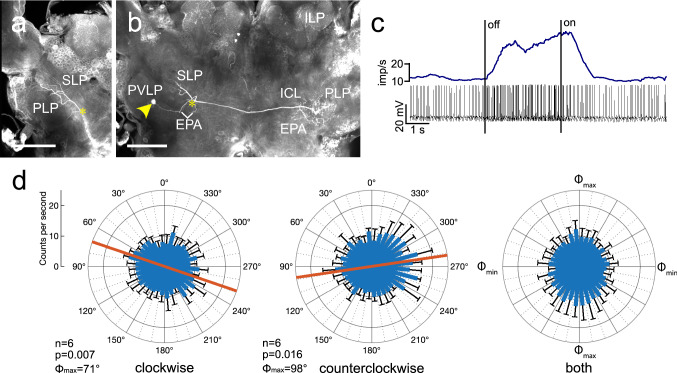
Morphology and physiology of the ILP interneuron, connecting the lateral protocerebrum of both brain hemispheres. **a**, Anterior and **b**, posterior stack of optical sections illustrating the morphology of the neuron, its connection is marked by yellow asterisks. Its soma lies in the soma rind between the antennal lobe and the anterior ventro-lateral protocerebrum of the brain (yellow arrowhead). Ramifications in the ipsilateral brain hemisphere are concentrated in the superior lateral protocerebrum (SLP), the epaulette (EPA), and the posterior lateral (PLP) and posterior ventro-lateral protocerebrum (PVLP). An axonal fiber projects contralaterally and gives rise to varicose ramifications in the inferior clamp (ICL), PLP and EPA. **c** Spike trains (bottom trace) and mean spike frequency (top, moving average with bin size of 0.5 s) illustrating inhibition of spiking during ventral light illumination (polarization angle at 0°). **d** Circular plots showing mean spiking activity (+ SD, black bars) plotted in 10° bins during clockwise rotation (left) and counterclockwise rotation of the polarizer (middle). Red bars indicate preferred polarization angles. Throughout both rotation directions spiking activity of the neuron is strongly reduced compared to background activity in the dark (25.7 imp s^−1^, not shown). Diagram on the right shows mean activities (+ SD) from all rotations with *Φ*_max_ set at 0°. Scale bars = 200 µm

A neuron with ramifications in several lateral protocerebral brain areas, termed interneuron of the lateral protocerebrum (ILP) was studied here for the first time (Fig. 10). The soma of the ILP neuron was located in the cell body rind between the antennal lobe and the anterior ventro-lateral protocerebrum (Fig. 10b). The neuron had wide, presumably dendritic, arborizations in the superior lateral protocerebrum, posterior lateral and ventro-lateral protocerebrum, and the epaulette in the ipsilateral hemisphere and sent an axonal process through the great commissure to wide ramifications concentrated in the posterior lateral protocerebrum, epaulette, and inferior clamp (Fig. 10a, b). The background activity of the ILP neuron was 24.5 ± 3.7 (SD) imp s^−1^. The neuron was markedly inhibited to 9.5 imp s^−1^ by ventrally presented blue light with a polarization angle of 0° (Fig. 10c) and, likewise, at all polarizer orientations during polarizer rotation (Fig. 10d). The neuron showed a significant response to polarization angle during both rotation directions. During clockwise rotation, the preferred *Φ*_max_ was 71° (Fig. 10d), during counterclockwise rotation, *Φ*_max_ was 98° (Fig. 10d), resulting in Δ*Φ*_max_ of –27°.

### Descending neurons

**Fig. 11:**
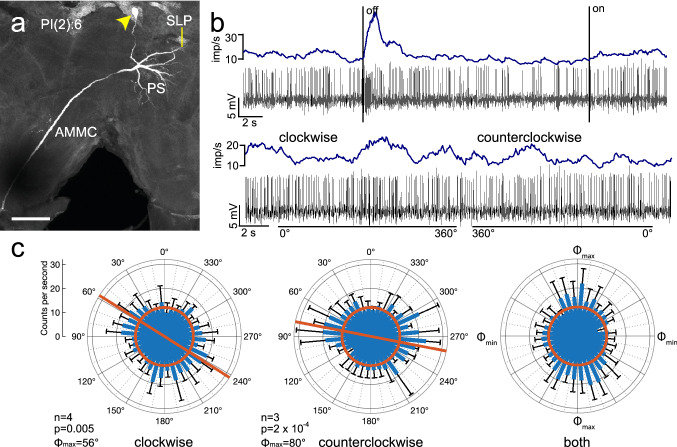
Morphology and physiology of a PI(2):6 descending neuron of the brain. **a** Stack of optical sections showing the morphology of the neuron. It has wide ramifications in the ipsilateral posterior slope (PS) and superior lateral protocerebrum (SLP), finely beaded processes in the contralateral antennal mechanosensory and motor center (AMMC), and an axonal fiber descending via the contralateral circumesophageal connective. Yellow arrowhead indicates the cell body of the neuron. **b** Spike trains (bottom traces) and mean spike frequency (top, moving average with bin size of 0.5 s) illustrating changes in spiking activity when ventral light (polarization angle at 0°) is turned off and on (upper part) and during clockwise and counterclockwise rotation of the polarizer (bottom). **c** Circular plots showing mean spiking activity (+ SD, black bars) plotted in 10° bins during clockwise rotation (left) and counterclockwise rotation of the polarizer (middle). The neuron shows increased spiking activity during polarizer rotation in both directions. Red bars indicate preferred polarization angles. Diagram on the right shows mean activities (+ SD) from all rotations with *Φ*_max_ set at 0°. Red circles indicate background activity in the dark. Scale bar = 100 µm

In five recordings descending neurons were labeled. These included 3 contralaterally descending neurons from the PI(2) group (one PI(2):5 and two PI(2):6 neurons), morphologically described first by Williams (1975) and two ipsilateral descending neurons termed IDN1 and IDN2. The PI(2):5 neuron did not show polarization angle-dependent modulation during rotation of the polarizer (Table 1). The somata of the two PI(2):6 neurons were located in the posterior pars intercerebralis. The neurons had wide arborizations in the ipsilateral posterior slope (Fig. 11a). A secondary area of slightly beaded processes was in the contralateral antennal mechanosensory and motor center (Fig. 11a). The background activities of the neurons were 11.1 ± 2.9 (SD) imp s^−1^ (Fig. 11c), and 11.2 ± 10 (SD) imp s^−1^. The neurons showed a phasic increase in spiking activity after the end of ventral light stimulation (Fig. 11b). Both recordings from PI(2):6 showed an angle of polarization dependent modulation of spiking rate (Fig. 11b, c), consisting of strongest excitation at *Φ*_max_ but no inhibition at *Φ*_min_ (Fig. 11c). The preferred angle of polarization (*Φ*_max_) in one recording was at 96° during clockwise rotation (Table 1) and at 119° during counterclockwise rotation (Δ*Φ*_max_ = − 23°; Table 1). The second cell had a preferred polarizer orientation at 56° during clockwise rotation and at 80° during counterclockwise rotation of the polarizer (Fig. 11c) resulting in a Δ*Φ*_max_ of − 24°.

**Fig. 12:**
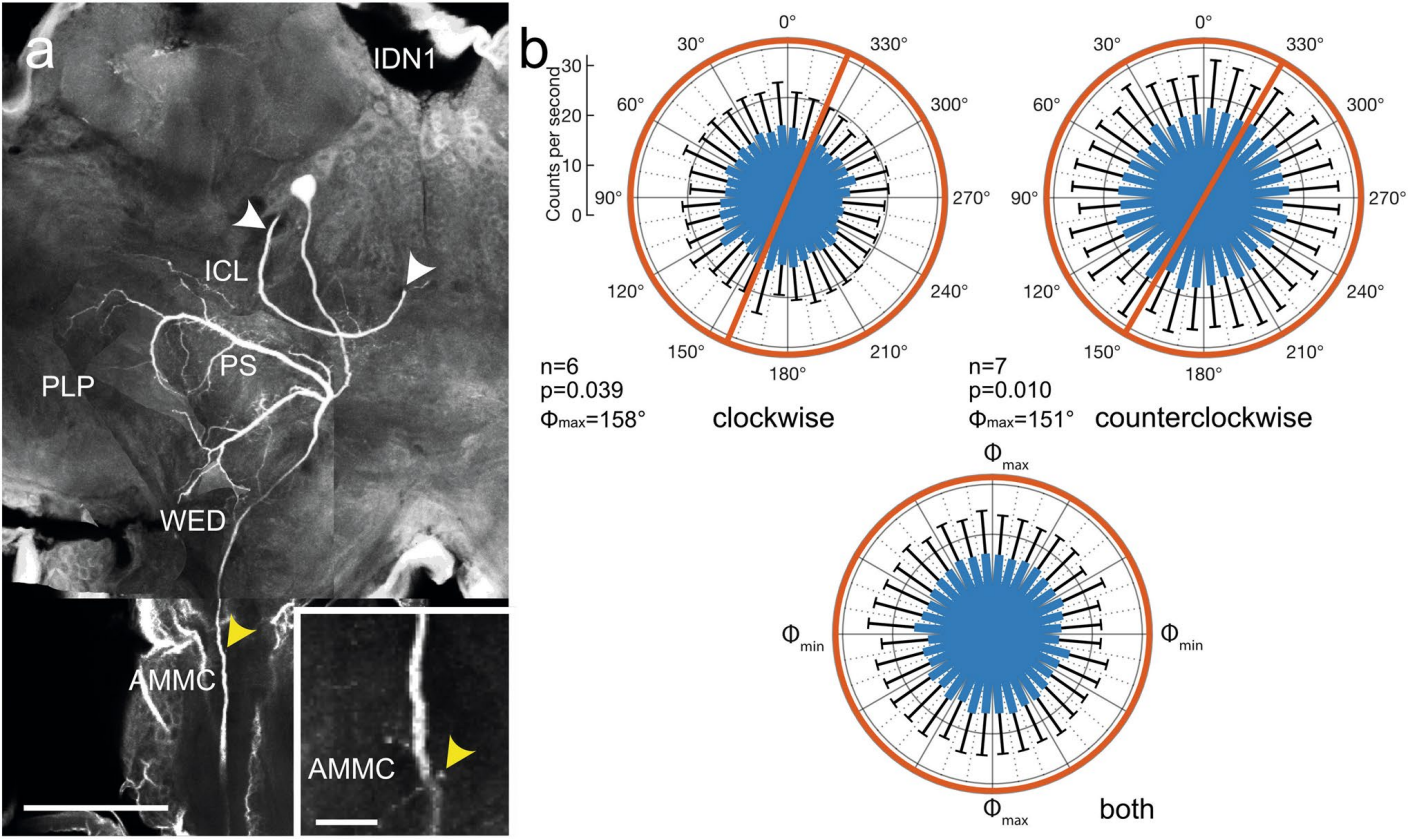
Morphology and physiology of the ipsilaterally descending neuron 1 (IDN1) of the brain. **a** Composite image of several stacks of optical sections showing the morphology of IDN1. The neuron has wide ramifications in the ipsilateral posterior slope (PS), inferior clamp (ICL), posterior lateral protocerebrum (PLP), and wedge (WED) and an axonal fiber descending via the ipsilateral circumesophageal connective. White arrowheads point to a fiber of a second colabeled neuron. Inset shows sparse beaded side branches in the antennal mechanosensory and motor center (AMMC, yellow arrowheads). **b** Circular plots showing mean spiking activity (+ SD, black bars) plotted in 10° bins during clockwise rotation (top left) and counterclockwise rotation of the polarizer (top right). Red circles indicate background activity in the dark. Spiking activity of the neuron, inhibited throughout polarizer rotation, shows polarization-angle dependent modulation during polarizer rotation in both directions. Red bars indicate preferred polarization angles. Bottom diagram shows mean activities (+ SD) from all rotations with *Φ*_max_ set at 0°. Scale bars = 200 µm (**a**), 25 µm (inset)

**Fig. 13:**
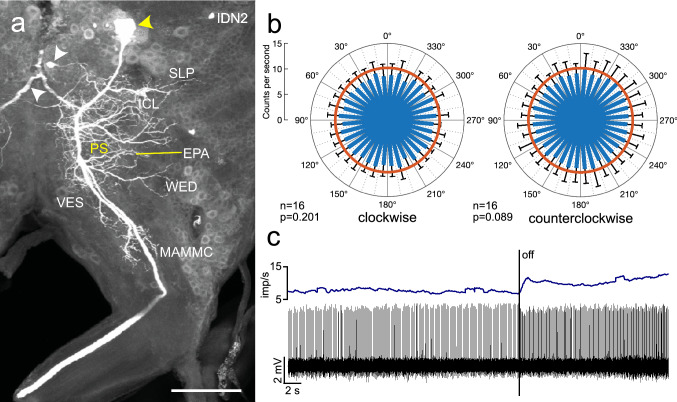
Morphology and physiology of the ipsilaterally descending neuron IDN2. **a** Stack of optical sections showing the morphology of the neuron. It has wide ramifications concentrated in the ipsilateral posterior slope (PS). Processes extend to adjacent areas including the superior lateral protocerebrum (SLP), the epaulette (EPA), the inferior clamp (ICL), the wedge (WED), the vest (VES), and the medial antennal mechanosensory and motor center (MAMMC). Yellow arrowhead points at the soma of the neuron, white arrowheads point at parts of other, colabeled neurons. **b** Circular plots showing mean spiking activity (+ SD, black bars) plotted in 10° bins during clockwise rotation (left) and counterclockwise rotation of the polarizer (right). Red circles indicate background activity in the dark. Neural activity shows no polarization*-*angle dependent modulation during polarizer rotation. **c** Spike train (bottom traces) and mean spike frequency (top, moving average with bin size of 0.5 s) illustrating changes in spiking activity when ventral light (polarization angle at 0°) is turned off. Scale bar = 200 µm

The ipsilaterally descending neurons IDN1 and IDN2 had a highly similar cell body position in the posterior lateral pars intercerebralis but differed in morphology of their dendritic side branches in the brain (Figs. 12, 13). Whereas both neurons had fine arborizations in the inferior clamp, posterior slope, and the antennal mechanosensory and motor center, the ramifications of IDN2 extended into several adjacent brain areas and differed in the pattern of side branches from those of IDN1. The background activity of IDN1 (30.6 ± 0.0 (SD) imp s^−1^) was substantially higher than that of IDN2 (10.8 ± 3.9 imp s^−1^). Spiking activity in IDN1 was markedly inhibited by ventral blue light (Fig. 12b) while inhibition was more moderate in IDN2 (Fig. 13b,c). Neural activity of IDN1 was significantly modulated by polarization angle during rotation of the polarizer in both direction (Fig. 12b), resulting in *Φ*_max_ values of 158° during clockwise rotation and 151° during counterclockwise rotation of the polarizer and a Δ *Φ*_max_ of 7°. In contrast, no polarization angle dependent modulation of activity occurred in IDN2 (Fig. 13b).

## Discussion

**Fig. 14:**
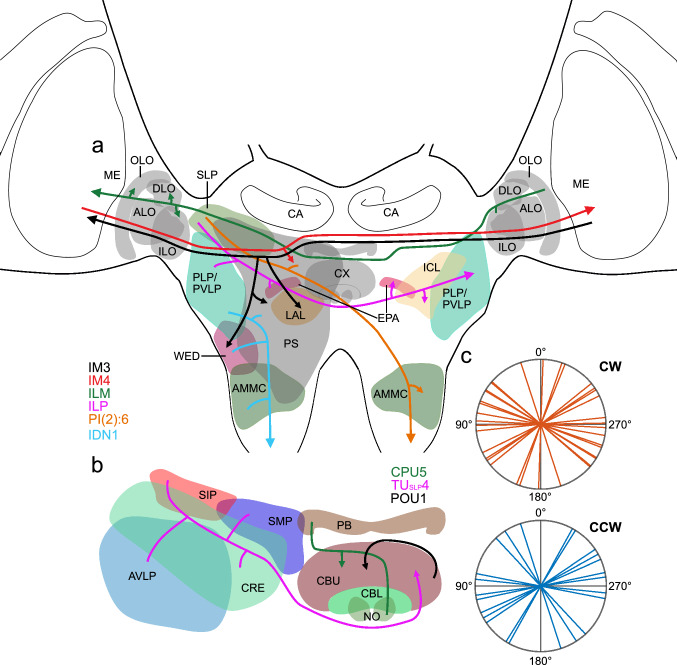
Summary diagrams illustrating areas in the locust brain innervated by neurons sensitive to polarized light from ventral direction (**a**, **b**) and preferred angles of polarization in those neurons (**c**). **a** Brain areas innervated by the different cell types excluding neurons of the central complex (CX), **b** neurons innervating the CX. Arrowheads point at presumed output areas of the neurons, blunt endings to presumed input sites. Only neurons sensitive to the angle of polarization during clockwise and counterclockwise rotation of the polarizer are included. **c** Circular plots showing mean *Φ*_max_ values from all neurons studied during clockwise (CW) and counterclockwise (CCW) rotation of the polarizer. Rayleigh tests did not reveal evidence for a clustering of *Φ*_max_ values at a particular angle (CW: *p* = 0.057; CCW: *p* = 0.103). ALO, anterior lobe of the lobula; AMMC, antennal mechanosensory and motor center; AVLP, anterior ventro-lateral protocerebrum; CA, calyx of the mushroom body; CBL, lower division of the central body; CBU, upper division of the central body; CPU5, type 5 columnar neuron of the PB and CPU: CRE, crepine; DLO, dorsal lobe of the lobula; EPA, epaulette; ICL, inferior clamp; IDN1, ipsilaterally descending neuron 1; ILM commissural interneuron of the LO and ME; ILO, inner lobe of the lobula; ILP, interneuron of the lateral protocerebrum; IM3, IM4, type 3, resp. 4 intermedulla neuron; LAL, lateral accessory lobe; ME, medulla; NO, noduli; OLO, outer lobe of the lobula; PB, protocerebral bridge; PI(2):6, descending neuron with soma in the pars intercerebralis; PLP, posterior lateral protocerebrum; POU1, type 1 pontine neurons of the CBU; PVLP, posterior ventro-lateral protocerebrum; PS, posterior slope; SIP, superior intermediate protocerebrum; SLP, superior lateral protocerebrum; SMP, superior medial protocerebrum; TU_SLP_4, type 4 tangential neuron of the CBU with cell body near the SLP; WED, wedge

Sensitivity to the polarization angle of light reflected from surfaces has been shown behaviorally and in photoreceptors in a number of insect species (Heinloth et al. 2018), but no information on central processing in the insect nervous system has been available to date. We have studied polarization sensitivity to blue light from ventral direction in neurons of the locust brain after painting the DRAs of both eyes black. Our study extends research by Beetz et al. (2016) who showed that certain optic-lobe neurons of the locust maintain sensitivity to polarization angle of light presented from lateral direction after the DRAs had been painted black. Our data reveal that certain interneurons of the optic lobe, CX, central brain, and two types of descending neurons are sensitive to polarization angle (Fig. 14), indicating that polarization sensitivity of photoreceptors in the main eye is processed centrally and may account for behavioral responses to non-celestial polarized light (Shashar et al. 2005).

### Properties of non-celestial polarization sensitivity in the locust brain

From a total of 43 recordings from neurons in the central brain of the locust, 11 neurons showed polarization-angle dependent modulation of spiking during bidirectional stimulation with polarized light from ventral direction (Table 1; Fig. 14a, b). The response amplitude in most neurons (vector strength *|r|*< 0.1) was relatively weak (Table 1) compared to values well above 0.2 in most neurons of the sky compass system (Bockhorst and Homberg 2015). Several factors might have contributed to this. One likely reason is the difference in acceptance angle of ommatidia in the DRA (~ 33°) vs. ommatidia in the main eye (~ 2°) combined with large overlap of receptive fields of adjacent ommatidia in the DRA but less so in the main eye (Schmeling et al. 2015). The small size stimulus as used here (visual angle 3.4°) and in previous studies would, therefore, stimulate photoreceptors in a substantially larger number of DRA ommatidia than in ventral eye ommatidia, where only small groups of ommatidia in the binocular zone of both eyes (Krapp and Gabbiani 2005) were likely stimulated. A second factor is the difference in polarization sensitivity (PS = 1.3–3.8 in the main eye) compared to PS values of up to 20 or more in DRA photoreceptors (Schmeling et al. 2014), resulting from high alignment of microvilli in the DRA but not in other regions of the eye. We, therefore, expect that polarization-angle dependent modulation of spiking becomes more pronounced when the locust views large fields of reflected polarized light such as provided by the surface of bodies of water (Shashar et al. 2005). This view fits well with anatomical and behavioral data from *Drosophila* (Wernet et al. 2012), where photoreceptors with low polarization sensitivity were found in the ventral half of the eye, apparently providing the retinal substrate for detecting large reflecting surfaces. Whether in locusts, like in horseflies (Meglič et al. 2019) and papilionid butterflies (Kelber et al. 2001), interactions of chromatically different photoreceptor types contribute to polarization sensitivity, remains to be seen.

In many neurons (IM3, IM4, CPU5, POU1, ILP, IDN1) polarization sensitivity occurred on the background of a strong response to ventral illumination irrespective of polarization angle. These responses to ventral light were usually long lasting, inhibitory or excitatory, but have not been explored any further for responsiveness to more specific features like chromatic preferences or others. In the sky-compass system, strong tonic inhibitory or excitatory responses to unpolarized blue light have, likewise, been encountered in the locust (Pfeiffer et al. 2011; Hensgen et al. 2022) and were partly explained by an imbalance in input from orthogonal analyzers of polarization angle. Moreover, polarization sensitivity in the sky-compass system is centrally combined with other aspects of vision, especially the azimuth of an unpolarized light spot (likely interpreted as the sun) resulting in increasing robustness of sky-compass signaling (Pegel et al. 2018, 2019; Zittrell et al. 2020; Takahashi et al. 2022).

In contrast to neurons of the sky-compass system, the neurons studied here did not show polarization opponency, i.e., excitation at *Φ*_max_ and inhibition at *Φ*_min_. Instead neurons were merely excited by polarization angles around *Φ*_max_ (TU_SLP_4, POU1, PI(2):6) or inhibited by angles around *Φ*_min_ (ILM) illustrating that inhibitory interactions between channels with orthogonal polarization-angle preferences might not occur.

Except for the TU_SLP_4 neuron, the preferred polarization angles differed by 6° (POU1) up to 33° (IM4, CPU5) when comparing the values for clockwise and counterclockwise rotation of the polarizer. This has also been observed in neurons of the sky-compass system (Träger and Homberg 2011; Bockhorst and Homberg 2015) and, likewise in neurons sensitive to polarization angle of light presented from low lateral elevations (Beetz et al. 2016). Higher angular *Φ*_max_ values during clockwise than counterclockwise rotation can be explained by response latencies (shown here as positive values) but the opposite shift which is more often observed (shown here as negative values) indicates an anticipatory shift which is more difficult to explain but may be based on adaptation effects during polarizer rotation (Bockhorst and Homberg 2015; Beetz et al. 2016).

### Neurons sensitive to polarized light from ventral direction

Only a small fraction of 11 out of 43 neurons studied here were sensitive to the polarization angle of ventrally presented light and showed similar and significant polarization angle dependent modulation of spiking during both turning directions of the polarizer (Fig. 14a, b). These included neurons interconnecting the two optic lobes, a neuron of the central brain, certain descending neurons, and a few cell types of the CX (Table 1). Cell types of the CX that in previous studies were consistently sensitive to zenithal polarization angle, TL2-, CL1-, CPU1a, and TB neurons were conspicuously unresponsive or showed weak polarization-angle dependent modulation of spiking only during one direction of polarizer rotation with vector strength values close to or below 0.1, suggesting that neurons of the sky-compass network in the CX are not involved in processing polarized light information from ventral direction (Table 1). Two polarization-sensitive neurons interconnecting the right and left medulla, IM3 and IM4, were morphologically highly similar to two IM neurons studied by Beetz et al. (2016) that were sensitive to the angle of polarization at low stimulus elevations but not to stimulation from the zenith. All IM neurons had wide, apparently presynaptic, arborizations in the posterior slope and surrounding brain areas suggesting that they are members of the same class of intermedulla neurons, sensitive to non-celestial polarized light. The lack of polarization sensitivity to counterclockwise polarizer rotation in the recordings from one IM4 and one ILM neuron may be related to different physiological states of the animal reflected by differences in background spiking activity and to the low number of polarizer rotations tested in these neurons (Table 1). Beetz et al. (2016) reported on a number of additional neurons projecting from the optic lobe to the lateral protocerebrum that were still polarization-sensitive after occluding the DRA. Because our electrodes were aimed at the CX and surrounding brain areas, those or similar neurons were not encountered in the present study. However, the polarization-sensitive commissural ILP interneuron, connecting lateral protocerebral brain areas across the brain midline, might receive input from optic lobe projection neurons studied by Beetz et al. (2016).

Although most CX neurons were completely unresponsive to polarization angle in our study, members of three cell types, all with ramifications in the CBU, were polarization-sensitive during both polarizer rotations (Fig. 14b). All of these neurons, the TU_SLP_4 tangential neuron, POU1 pontine neuron, and CPU5 columnar neuron, may be interconnected in the CBU and have not been studied physiologically before. In contrast to most neurons outside the CX, the response amplitudes of these had vector strength values between 0.11 and 0.38 (Table 1), similar to values found in CX neurons of the sky compass system (Bockhorst and Homberg 2015). Notably, only single recordings from CPU5 and POU1 neurons showed sensitivity to polarization angle during both directions of polarizer rotation. Recordings from a second CPU5 neuron were unresponsive and from a second POU1 neuron only responsive during counterclockwise rotation of the polarizer (Table 1). Similar cases of polarization sensitive and insensitive neurons of the same morphological type have also been observed in the CX when stimulating with zenithal polarized light (Heinze and Homberg 2009) and may indicate that responsiveness in these neurons depends on the physiological state of the animal such as feeding state, mating state, or different levels of alertness. As a possible indicator of this, the background activities in the responsive and unresponsive CPU5- and POU1 neurons differed substantially. Alternatively, morphologically similar cell types may be functionally distinct as shown for different subtypes of tangential ring neurons in the fly *Drosophila* (Hardcastle et al. 2021). The preference of ring neurons innervating the superior bulb for horizontally polarized light has prompted Hardcastle et al. (2021) to suggest that these neurons may provide polarization input to the CX from ventral directions, but physiological evidence for this assumption is still lacking. Given the role of the upper division of the central body in context dependent changes of steering commands (Shiozaki et al. 2020), sensitivity of neurons in this brain area to polarized light from ventral directions might specifically be suited to modify goal directions in locusts when flying over extended bodies of water as has been shown by Shashar et al. (2005). Taken together, the data suggest that a distinct pathway for ventral polarization sensitivity into the CX exists, but whether signaling via these neurons results in a compass-like representation of non-celestial polarization angles in the CBU, awaits further experimental evidence.

Robust polarization sensitivity was, finally, found in two types of descending neurons, the contralaterally descending neuron PI(2):6 (Williams 1975) and an ipsilateral descending neuron termed IDN1. The PI(2):6 neuron has been studied before by Träger and Homberg (2011). The authors showed that the neuron is polarization-sensitive when stimulating the animal from dorsal direction. As found here and by Träger and Homberg (2011), the neuron was not inhibited at *Φ*_min_ and showed tuning to different polarization angles, which in Träger and Homberg (2011) systematically varied with time of day of the recording, suggesting a role in time compensated sky-compass navigation. In view of the data presented here, it might be worth to reinvestigate, whether the PI(2):6 neuron receives input from the DRA at all, or not just from all other ommatidia of the eye including those facing the sky. The neuron, might, therefore, signal deviations from a straight course based on polarization input from below (water surface) or above (sky). The IDN1 and 2 neurons appear to be members of a group of ipsilaterally descending neurons with highly similar main fiber course but distinct patterns of side branches in the brain (FFDN1 neuron: Baader et al. 1992; PDDSMD neuron: Rind 1990; ipsilaterally descending neuron: Träger and Homberg 2011). These neurons have been reported to be sensitive to dorsally presented polarized light (Träger and Homberg 2011), yaw rotation (Rind 1990), and to progressive optic flow, but not to yaw (Baader et al. 1992). The neurons, therefore, clearly differ in sensory input, but may, like the PI(2):6 neuron, be involved in various aspects of flight balance and direction maintenance.

### A neural network for non-celestial polarized light processing in the locust brain

The present study together with previous work by Beetz et al. (2016) provides a first but still highly incomplete skeleton of a neural network processing non-celestial polarized light in the brain of the locust, and possibly other insects as well (Fig. 14a, b). Polarization-angle dependent responses of blue photoreceptors of the main retina are passed via unknown interneurons converging onto large-field medulla interneurons, such as intermedulla neurons reported here and/or medulla projection neurons reported by Beetz et al. (2016). Several of these neurons share varicose outputs in the posterior slope with dendritic inputs of descending neurons (Fig. 14a) suggesting direct synaptic contact. Higher-order processing in the brain, possibly involving the mushroom bodies and, as shown here, the central body (Figs. 6, 7, 9, 14a, b), might, in addition, be involved in more specific goal-directed responses, such as selection of feeding sites, roosting sites, or egg-laying places, depending on vegetation and internal needs. Although this study can only provide a first and highly incomplete insight into processing pathways for non-celestial polarized light detection, it might serve to guide future studies to more specifically investigate neurons and brain areas for processing of polarization signals from sources other than the sky.

## Data Availability

All data that support the findings of this study are available from the corresponding author.

## Abbreviations

CBL: Lower division of the central body
CBU: Upper division of the central body
CX: Central complex
DRA: Dorsal rim area
PB: Protocerebral bridge
